# The Evaluation of a Long-Term Experiment on the Relationships between Weather, Nitrogen Fertilization, Preceding Crop, and Winter Wheat Grain Yield on Cambisol

**DOI:** 10.3390/plants13060802

**Published:** 2024-03-12

**Authors:** Lukáš Hlisnikovský, Ladislav Menšík, Muhammad Roman, Eva Kunzová

**Affiliations:** 1 Department of Nutrition Management, Crop Research Institute, Drnovská 507, Ruzyně, 161 01 Prague, Czech Republic; ladislav.mensik@vurv.cz (L.M.); kunzova@vurv.cz (E.K.); 2 Department of Environment, Faculty of Environment, Jan Evangelista Purkyně University in Ústí nad Labem, Pasteurova 15, 400 96 Ústí nad Labem, Czech Republic; m.maan26@outlook.com

**Keywords:** air temperature, correlation, crop rotation, mineral N, precipitation, linear plateau model, *Triticum aestivum* L.

## Abstract

In this paper, a sequence (1979–2022) of a long-term trial established in Lukavec in 1956 (Czech Republic) focusing on the effect of weather, various nitrogen (N) fertilization methods (control, PK, N1PK, N2PK, and N3PK) and preceding crops (cereals, legumes, and oil plants) on winter wheat grain yield is presented. The weather significantly changed at the site of the long-term trial. While the trend in the mean temperature significantly increased, precipitation did not change significantly over the long term. Four relationships between weather and grain yield were evaluated to be significant: (a) the mean temperature in February (r = −0.4) and the precipitation in (b) February (r = −0.4), (c) March (r = −0.4), and (d) May (r = 0.5). The yield trends for all the fertilizer treatments increased, including the unfertilized control. The N3PK treatment provided the highest mean grain yields, while the unfertilized control had the lowest yields. Comparing the preceding crops, the highest yields were harvested when the wheat followed the legumes. On the other hand, the cereals were evaluated as the least suitable preceding crop in terms of grain yield. According to the linear-plateau model, the optimal nitrogen (N) dose for modern wheat varieties, following legumes and under the trial’s soil climate conditions, was 131 kg ha^−1^ N, corresponding to a mean grain yield of 8.2 t ha^−1^.

## 1. Introduction

Mineral fertilization is the cornerstone of conventional crop production. Their regular application to the soil is essential for modern agriculture and enables the current level of crop yields to be maintained. The most important nutrient we supply to the soil in the form of mineral fertilizers is mineral nitrogen (N). Awareness of the importance of N for living organisms dates back to the 18th century [1]. In the mid-19th century, experiments in Rothamsted in the United Kingdom showed that the application of N fertilizers significantly increased the yield of crops. There were three natural sources of N that could be applied to agricultural land at that time. Ammonium sulfate, which was produced during the distillation of coal, Guano sediments from South America, and finally, Chilean nitrate [2]. Unfortunately, none of these resources were sustainable for the future development of the human population on Earth as the sources were limited. For this reason, people focused on airborne N and began to discover how to convert it into a form that could be applied to farmland. The processes discovered in Germany in 1898 and in Norway in 1905 looked promising, but the procedures were costly [3,4]. The real breakthrough was also discovered in Germany a little bit later, where the cooperation among Mr. Haber, Mr. Bosh, and people from the BASF company led to the opening of a factory in 1913, producing N derived from the Haber-Bosh process [4]. The main purpose of this process was primarily seen in the military industry and only after the end of the second world war did it find its application in agriculture [1], resulting in the so-called green revolution (together with the development of plant genetics [5]). Since then, the yield of wheat grain [6,7] and the consumption of N fertilizers [8,9,10,11] have been increasing worldwide. However, there has been a recent stagnation of wheat yields in some areas [12,13].

Nitrogen is the most important nutrient for wheat and other crops, directly influencing wheat yield and quality [14,15,16]. It can also have an indirect impact through factors such as chlorophyll content [17] and root density [18]. Applying insufficient amounts of mineral N will not meet the requirements of modern wheat varieties, leading to decreased yields. On the other hand, excessively high doses are undesirable as they can damage the environment [19], degrade soil properties [20], be inefficient in terms of finances [21], and even decrease production [22,23]. For these reasons, it is desirable to optimize the N rates. Fertilizer optimization is site-specific, as yields are primarily influenced by natural factors such as soil and climatic conditions in a specific location. Therefore, the recommendation for one area may be completely different from that of another area with a different soil type or weather pattern. The determination of the optimum N rate can be achieved using several response models that compare grain yield as a function of the applied N rate. These include, for example, quadratic, linear plateau, quadratic plateau, or logistic models [24]. The basic assumption is to design the trial in such a way that the response of wheat to increasing doses of mineral N is typical. In other words, the yields should gradually increase with N doses until they reach the maximum function and then begin to decrease (concave downward function). This decrease in grain yield at excessive mineral N rates, which is typical for cereals, may be attributed to lodging in large-scale fields. However, the lodging of individual treatments with high mineral N rates is not common in small-plot experiments. Declining yields caused by high mineral N rates (in fields or trials) are due to the metabolic processes in wheat [25]. This consequence is subsequently exploited for optimization.

Along with fertilization, weather also plays an important role in the production of wheat grain yield [26,27]. The first scientific papers that focused on the relationship between weather and crop yields date back to the turn of the 19th and 20th centuries [28,29,30,31] and continue to this day. Recently, many articles analyzing the links between weather and cereal yield have been published [32,33,34,35], concluding that different weather components, such as precipitation and temperatures in individual months, play a significant role in the formation of yield. Analyses between weather and crop production are particularly important because of ongoing climate change. Analyses of the relationship between weather and crop production are particularly important due to ongoing climate change. Defining climate change is relatively challenging [36] and is interpreted differently by various societies [37]. In general terms, climate change can be described as a long-term shift in climatic conditions characterized by high inter-annual variability in weather. Climate change is manifested by a gradual warming of all the components of the environment (air, soil, and water) and was recorded worldwide [38,39,40,41,42,43,44,45]. Warming is also influencing the distribution of precipitation [46,47,48]. Climate change thus poses a significant challenge to agriculture that can be partially mitigated by time of sowing and choice of wheat varieties [49] and changes in crop species, land allocation, irrigation, or fertilization [50]. However, climate change is not always associated with negative effects, and adaptation opens up new opportunities, especially in agriculture [51,52,53].

Last but not least, the yields of cereals are significantly influenced by crop rotation [54] or, more precisely, by the preceding crop. The preceding crop influences the physical, biological, and chemical properties of the soil in which the wheat is subsequently planted. The nutrient requirements and preferences of the preceding crop, the way it is fertilized, and the proportion of the same or similar crops (such as cereals) in the crop rotation have an impact on the nutrient supply in the soil, the occurrence of pathogenic organisms, and ultimately grain yield [54,55,56]. A comparison between soybean and winter rape showed that soybean is a more suitable preceding crop for wheat than winter rape, significantly increasing the grain and straw yield of wheat [57]. Unfortunately, in the Czech Republic, winter rape is the dominant crop preceding wheat due to the high demand from the food and energy industries. The least suitable preceding crop for wheat is wheat itself, followed by other cereals such as oats. On the other hand, field peas, faba beans, chickpeas, lentils, and lupins are preceding crops that have a beneficial influence on wheat grain yield [58].

In this paper, we evaluated the development of weather (mean temperature and precipitation) at the trial site, the effect of weather on winter wheat grain yield, and the effect of different N fertilizer treatments and preceding crops on wheat grain yield. The data comes from a long-term experiment in the Czech Republic that was established in 1956 on the Cambisol soil type. The paper also includes an analysis of the optimal mineral N rate for specific soil and climatic conditions utilizing the linear plateau response model.

## 2. Results

### 2.1. Weather Development

**Figure 1:**
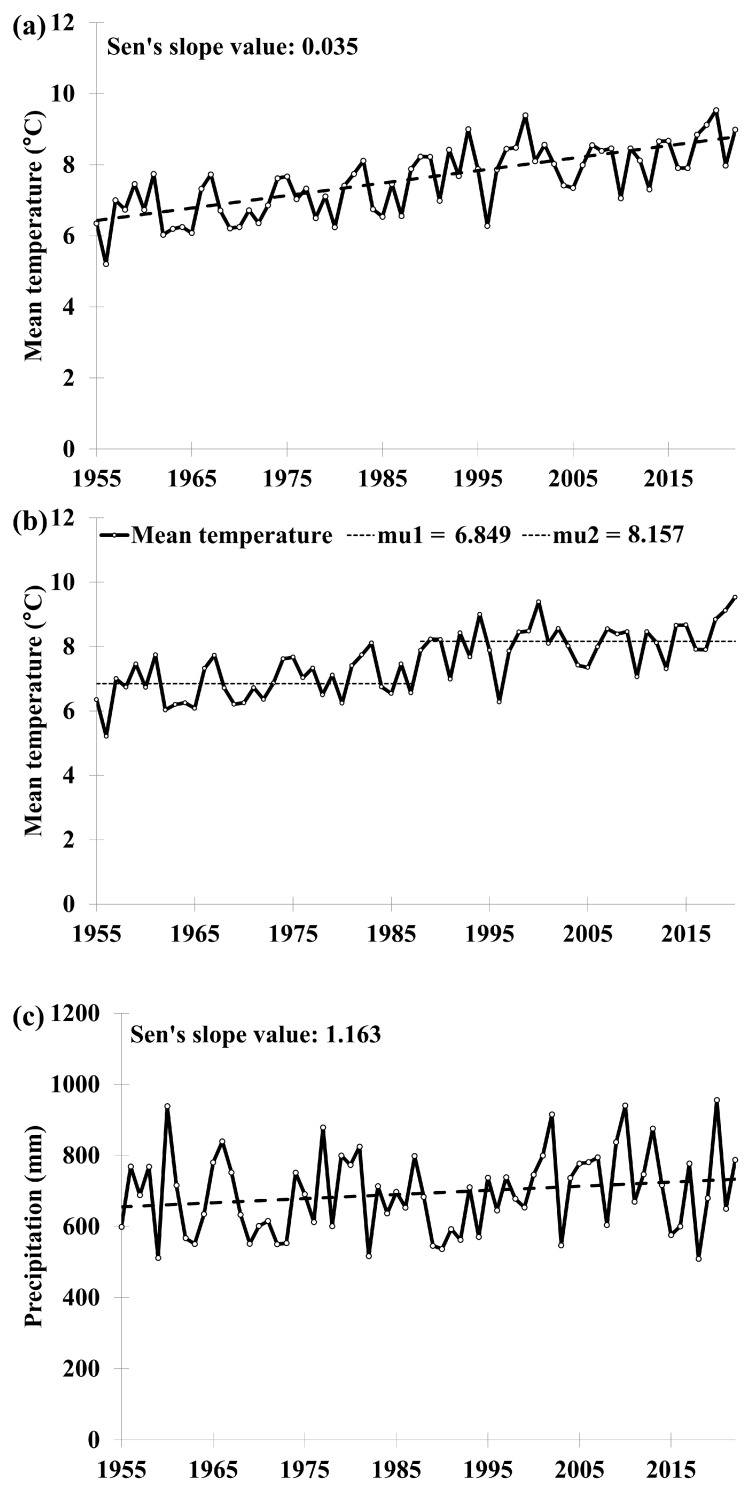
The annual development of (**a**) mean temperature (°C) and (**c**) precipitation (mm) at the Lukavec experimental station from 1955 to 2022. The Sen’s slope value indicates an annual increase of the parameter. (**b**) The separation of two time periods characterized by different mean temperatures (mu1; mu2; °C) based on a homogeneity test.

Since 1955, the long-term experimental site in Lukavec has been warming up. The average temperature has been increasing, and this trend is statistically significant (Figure 1a). The year that can be described as a turning point in terms of temperatures was determined, based on a homogeneity test, to be 1987. From 1955 to 1987, the average annual temperature was 6.849 °C, while the period after that year was characterized by an average temperature of 8.157 °C (Figure 1b). Precipitation has been slightly increasing (the Sen’s slope value is 1.163 mm year^−1^), but the trend is not statistically significant (Figure 1c). The results of the time series analysis of weather parameters indicate that the crops cultivated in Lukavec must adapt to the gradually rising average temperatures. However, they still receive a similar amount of rainfall as in the past.

### 2.2. The Relationship between Weather and Grain Yield

**Figure 2:**
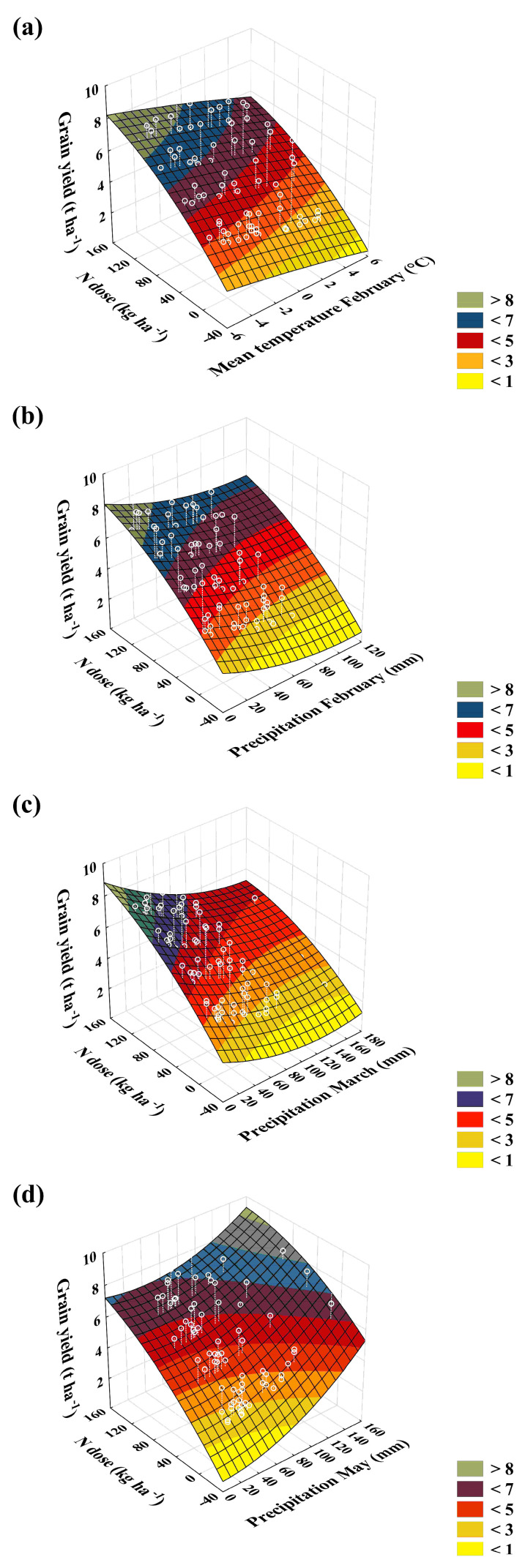
The relationship between grain yield (t ha^−1^, white dots), N dose (kg ha^−1^), and (**a**) mean temperature in February and (**b**) precipitation in February, (**c**) March, and (**d**) May. The color scale represents different yield levels (t ha^−1^).

According to the MANOVA results, wheat grain yields in Lukavec were influenced by weather only by 10% (d.f. = 27, F = 191, *p* < 0.001). The influence of temperature and precipitation on the yields in Lukavec could be partially assessed through correlation analysis, a method that was suggested and utilized in early studies examining the connection between weather and crop yields [59]. The correlation analysis used in this paper compared the relationship between the mean temperature and precipitation of all months during the growing season (October to July) and the mean grain yield. Based on the correlation analysis, four out of twenty relationships were confirmed as significant: (a) mean temperature in February (r = −0.4) and precipitation in (b) February (r = −0.4), (c) March (r = −0.4), and (d) May (r = 0.5). The results are shown in Figure 2.

As Figure 2a shows, higher wheat yields can be expected in Lukavec when February is cold, especially when the average temperature is below zero (−4 °C), and the yields decrease as the average temperature increases. At the same time, higher yields can be expected when February and March have lower precipitation levels (Figure 2b,c). Conversely, higher precipitation in May has a positive effect on yields (Figure 2d). Unfortunately, mean temperatures in February increase over time by 0.02 °C annually, as well as precipitation in February (0.02 mm) and March (0.1 mm), although insignificantly. In summary, colder and drier winters lead to higher yields in Lukavec, while warmer and wetter winters reduce yields. On the other hand, rainy Mays are associated with higher yields, and there is a growing trend of precipitation in May (0.07 mm annually), although it is not statistically significant.

### 2.3. Grain Yield Development and the Effect of Fertilizer Treatment on Grain Yield

**Figure 3:**
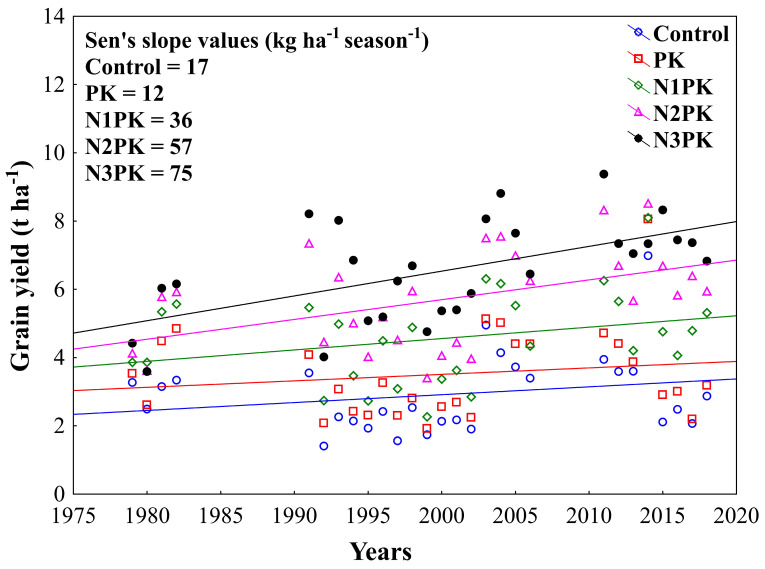
The development of grain yield (t ha^−1^) in the 1979–2018 period (*n* = 28 seasons) as affected by the fertilizer treatment (control, PK, N1PK, N2PK, and N3PK).

Winter wheat grain yields have an increasing trend in Lukavec in all fertilizer treatments, even in the unfertilized control. According to the Sen’s slope value (Figure 3), the lowest seasonal grain yield increase was recorded in the PK treatment, followed by the control and N1PK, N2PK, and N3PK treatments.

In comparison with the weather, fertilizer treatment represents a dominant factor influencing grain yield in Lukavec. Based on the MANOVA results, fertilization affected the grain yield by 90% (d.f. = 4, F = 1648, *p* < 0.001).

According to the non-parametric ANOVA results, winter wheat grain yields from the 1979–2018 and 2019–2022 periods were significantly affected by fertilizer treatments (*p* < 0.0001). The results are shown in Table 1.

The lowest yields were always recorded for the unfertilized control treatment. The application of mineral fertilizers was associated with an increase in yield for all fertilization treatments. In the seasons between 1979 and 2018, the period before the increase in mineral N doses (2019–2022), all fertilizer treatments were statistically significantly different from each other (Table 1, central column), and the yields increased linearly together with increasing N doses. Higher doses of mineral N in the 2019–2022 period resulted in statistically comparable yields between the treatments (Table 1, right column).

The second factor contributing to the increasing trends in grain yields is the development and introduction of modern wheat varieties. According to the MANOVA results, grain yields were influenced by the wheat variety by 25% (d.f. = 6, F = 95.4, *p* < 0.0001), while fertilization influenced the yields by 74% (d.f. = 4, F = 289.5, *p* < 0.0001).

The effect of the wheat variety on grain yield was statistically significant between 1979 and 2022 (Kruskal-Wallis test followed by the Conover-Iman post hoc procedure; *p* < 0.0001). The highest grain yields were recorded in the modern wheat varieties (Contra, Mulan, and Julie), while lower yields were provided by the older wheat varieties (Brea, Vega, Slávia, and Hana). The results are shown in Table 2.

### 2.4. N Dose Optimization

**Figure 4:**
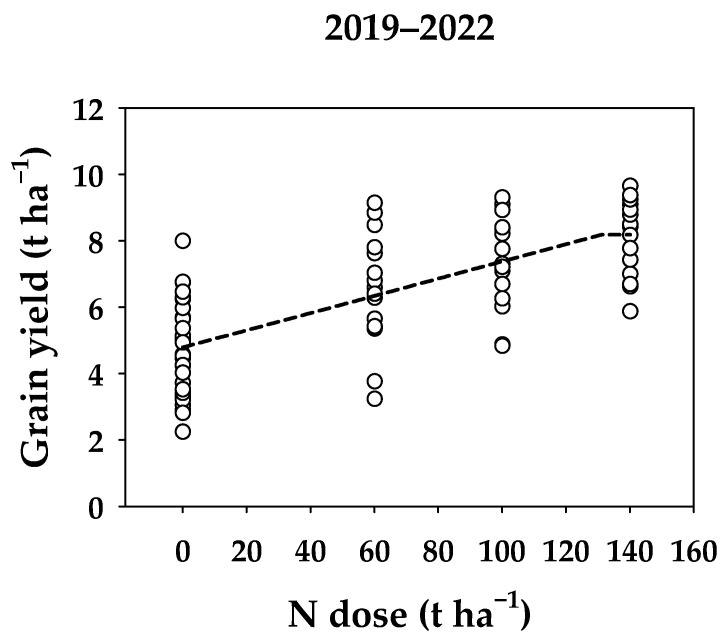
The relationship between wheat grain yield (t ha^−1^) and increasing N dose (kg ha^−1^) from 2019 to 2022. The mean grain yields (black circles) are interleaved with the linear plateau model (dashed line).

As the data shows, the determination of the optimum N dose for wheat could be based on a non-parametric ANOVA, resulting in the recommendation of N3PK treatment (based on the data from 1979 to 2018). However, this could be misleading, as wheat response to higher N doses (higher than 120 kg ha^−1^) could also be statistically significantly different from the N3PK treatment. That is why the mineral N doses from 2019 increased, as it was clear that the trial methodology was creating barriers to the full potential of wheat in Lukavec. The results of the methodology change are visible in Table 1 (right column). According to the post hoc analysis, the N2PK treatment yielded statistically comparable results to the N3PK treatment, enabling the use of a linear plateau model for determining the optimum N dose (Figure 4). The shoulder point was modeled at a rate of 131 kg ha^−1^ N, corresponding to an average yield of 8.2 t ha^−1^ when wheat follows legumes in a crop rotation.

### 2.5. Effect of the Preceding Crop

The effect of the preceding crop on wheat grain yield was found to be significant between 1979 and 2018 (Kruskal-Wallis test followed by the Conover-Iman post hoc procedure; *p* < 0.0001). The results are shown in Table 3.

## 3. Discussion

### 3.1. Weather Development

The warming effect observed from the long-term time series in Lukavec confirms the trend reported from the Czech Republic [38], surrounding countries, such as Poland [60], Slovakia [61], Austria [40], Germany [39], and worldwide [62]. This warming trend, called global warming, is already influencing ecosystems and the environment [63,64], and human activities, including agriculture [65], industry, transport and energy sectors [66], human health [67], and shaping old weather patterns, such as precipitation [68]. Rising air temperature adversely affects the soil water regime, increasing evapotranspiration. At the same time, warmer air has an increased capacity to hold water vapor. This results in a prolonged period without precipitation in the hottest months. This period is then replaced by the release of large amounts of accumulated precipitation. Both events are negative aspects for crop production, threatening crop development and growth and soil quantity and quality through wind and water erosion. However, not all changes and impacts are and will be negative. In agriculture specifically, global warming and the associated climate change are having both negative and positive impacts, depending on the location and plant species. From a global perspective, the crop yields of currently dominant crops in Italy and southern central Europe can be deteriorated by the current climate change [69]. In Australia, higher temperatures (>34 °C) can significantly reduce grain yield due to increased leaf senescence [70]. On the other hand, conditions in the UK and some parts of northern Europe are expected to be favorable for various crops [69]. In the Czech Republic and surrounding states, cereal yield can be expected to increase in the uplands as decreasing water availability and heat stress will occur more often and with greater intensity in the lowlands due to climate change [48,52,53,71]. Additionally, rising temperature is connected to the expansion of the vegetation zone, which is suitable for thermophilic crops from the lowlands to the uplands [51]. Thus, the combination of increasing temperatures and naturally higher precipitation in the uplands should result in more favorable conditions for wheat growing there in the future.

### 3.2. The Relationship between Weather and Grain Yield

The dominant factor affecting grain yield in Lukavec was fertilizer treatment (90%). For example, in a long-term trial in northeast Germany, fertilization influenced the yield of spring barley by only 11%, while it influenced the weather by 55% [35]. As mentioned in the results, a higher yield in Lukavec can be expected when winters are cold and dry, and lower yields are connected to warmer and wetter winters. Similar results describing the effect of a wetter spring on the development and yield of winter wheat were found in Rothamsted [34] and in a meta-analysis of trials running in the United Kingdom [72]. These studies concluded that wetter spring conditions could lead to N losses from the soil, resulting in the reduced availability of this important nutrient for wheat. Another explanation could be that mild winters facilitate the exceptional growth of the above-ground components of wheat, rendering them more susceptible to spring frosts. Finally, wetter spring conditions provide suitable circumstances for washing off protective sprays, preventing the entry of agricultural equipment to the field and promoting the development and increased pressure of pathogenic organisms [72]. May precipitation is correlated positively with grain yield in Lukavec. The same results were found in the USA, where yield reduction was correlated when April and May precipitation was limited in Oklahoma and May rainfall was low in Kansas [33].

### 3.3. Grain Yield Development and the Effect of Fertilizer Treatment on Grain Yield

In Lukavec, wheat responded very well to the mineral fertilizers applied to the soil. The lowest yields were recorded in the control treatment groups, and the yields increased linearly with increasing rates of mineral N. At the same time, all the treatments were statistically different from each other between 1979 and 2018. A similar pattern of dependence of grain yield on mineral N was previously observed in other long-term experiments in various countries, including the Czech Republic [73,74], Hungary [75], Germany [76], China [77], the USA [78], and the United Kingdom [79] showing the irreplaceable role of N in wheat nutrition. This is confirmed using the results of the PK treatment, which were statistically higher when compared to the control between 1979 and 2018. However, different results were found in the yields between 2019 and 2022, when the yields from the PK treatment were comparable with the control and N1PK treatment. The reason for statistical indifference in the later period was connected to the preceding crop. While three different preceding crops were evaluated in the 1979–2018 period, legumes only served as a preceding crop to wheat in the last period. This provided fixed aerial N to the wheat to all treatments, resulting in less distinct boundaries of statistical significance between the compared treatments.

The degree of response of wheat to the applied fertilizers is significantly influenced by the soil type at the site [80,81]. Cambisols are the most abundant soils in the Czech Republic [82] and are considered to be naturally less fertile than Chernozems. The lower fertility of Cambisols is evident in the significant increase in the cultivated crop yield when externally supplied with mineral N. In naturally fertile Chernozems, the differences in wheat yield between N treatments may be less pronounced [83,84]. Additionally, if a suitable preceding crop, such as legumes, is used, these differences in Chernozems may not occur at all [85].

As mentioned in the introduction, the increase in global wheat yields in the second half of the 20th century was attributed not only to the improved and more stable availability of mineral fertilizers but also to significant advancements in genetics. Particularly important was the discovery of the genes that led to a reduction in wheat stalk height. At the beginning of this breakthrough is the work of the Japanese scientist Gonjiro Inazuka, who was the originator of the wheat variety named Norin 10. This was characterized by the presence of the *Rht1* and *Rht2* genes (dwarf genes), which reduced the height of the wheat from over 150 cm to between 60 and 110 cm. Later, Norin 10 became a springboard for breeding new varieties in the USA and Mexico [86]. Wheat varieties containing these dwarf genes were better able to resist lodging and had a higher harvest index. The introduction of these genes also reduced the grain size and grain protein content [87]. However, the ability to apply higher rates of mineral N without losing part of the yield to lodging led to unprecedented gains in grain and protein. The successful implementation of dwarf genes in practice, along with the improved availability of mineral fertilizers, led to the so-called green revolution [88]. This revolution successfully addressed the growing need for food production in the second half of the 20th century. In this paper, we evaluated only short-straw wheat varieties that were introduced in a long-term trial in the early 1980s. However, we know from previous evaluations that the transition from long to short-straw varieties led to substantial yield increases [73,74], demonstrating the significant contribution of modern wheat varieties. Modern varieties are constantly evolving, adapting to changing climate conditions, improving their water management, nutrient utilization, and resistance to pathogens. The use of modern and up-to-date wheat varieties in the long-term trial has thus contributed to increasing yield trends. Recently, however, yield stagnation has been observed in crops such as wheat, barley, and oats in France [89] or in some areas worldwide [13]. This stagnation does not seem to be due to a lack of progress in the field of genetics but to other influences such as climate change, a reduction in cropping practices (especially a lower proportion of leguminous crops), or a decrease in fertilizer application due to political decisions [12,90].

### 3.4. N Dose Optimization

According to the linear plateau model, a reasonable dose of mineral N for wheat is 131 kg ha^−1^, corresponding with a grain yield of 8.2 t ha^−1^. In comparison to other areas in the Czech Republic, this recommended dosage is quite high. Similar studies have suggested a dosage of 107 kg ha^−1^ N, which corresponds to an average yield of 8.1 t ha^−1^ on the Chernozem soil type [85], and 44 kg ha^−1^ N, which corresponds to an average yield of 7.4 t ha^−1^ on the Luvisol soil type [91] when wheat follows legumes. This high value of the shoulder point is primarily due to the soil and climatic conditions of the site, i.e., less fertile soil and less suitable weather conditions. Compared to other locations, farmers on this land have to apply more fertilizers to achieve comparable yields to farmers in the lowlands and on more fertile soils.

### 3.5. Effect of the Preceding Crop

The crops included in the rotation have varying nutrient requirements, necessitate different amounts of nutrients, are susceptible to different pathogens, and are fertilized with various types of fertilizers. For this reason, the crops are divided into soil-deteriorating and soil-improving crops. For example, root crops are beneficial for soil improvement in terms of fertilization. They are traditionally fertilized with farmyard manure, which supplies organic matter to the soil. This is crucial for maintaining soil health. Manure also decreases inter-annual yield variability, acting as a factor that stabilizes yields [76]. Generally, the same crop is the least suitable preceding crop for the succeeding crop because it has identical nutrient requirements. Even supplying a sufficient amount of fertilizers may not ensure adequate yields when crops are cultivated in monocultures [92]. Another negative aspect of using the same crop as the preceding crop is the increased pressure from pathogenic organisms targeting that crop [56]. In our case, legumes were evaluated as a preceding crop that provided the highest yields. Similar results were found in [73,74,93,94,95]. Legumes are soil-improving crops due to their symbiotic relationship with rhizobacteria. These bacteria are able to fix airborne N in the soil, where it is used by both soil fauna and flora. This symbiosis benefits both the soil and the farmer by combining improved soil properties with reduced fertilizer costs [96,97]. Although legumes have benefits for all the parties involved, these benefits are not utilized well in current agriculture practices in the Czech Republic. The opposite is true: the area under legumes is declining due to the increased production of wheat and winter rape as well as a decrease in livestock numbers [98]. The low proportion of arable land covered by legumes is not only a problem in the Czech Republic but also in Europe [96,99].

## 4. Materials and Methods

### 4.1. The Long-Term Trial Description

The long-term field trial was established in Lukavec, located in the Czech Republic in central Europe, in 1956. The objective of the trial was to analyze the impact of various fertilizer treatments and weather conditions on the yield and quality of arable crops as well as the chemical properties of the soil. The GPS coordinates were N 49°57′ and E 14°99′. The elevation was 620 m asl. The soil type was Cambisol. The thickness of the arable layer was 0.25–0.30 m. The long-term mean annual temperature and precipitation were 7.5 °C and 694 mm from 1955 to 2020, respectively. According to the Köppen-Geiger climate classification [100], the trial site was located in a warm summer continental climate area (DfB).

The trial consists of four identical fields. Each field was divided into 48 plots, where 12 fertilizer treatments were analyzed using a completely randomized block design. Each treatment was repeated four times (12 × 4 = 48). The size of one plot was 8 × 8 m. To eliminate the edge effect, only the central area of the plot, measuring 5 × 5 m, was harvested and analyzed for experimental purposes. Five out of twelve fertilizer treatments were evaluated in this paper: (1) the control (unfertilized since trial establishment); (2) PK (mineral phosphorus (P) and potassium (K) without mineral N); (3) N1PK (mineral N, P, and K; N dose of 40 kg ha^−1^); (4) N2PK (N dose of 80 kg ha^−1^); and (5) N3PK (N dose of 120 kg ha^−1^). While mineral N was strictly applied in the specified doses, the doses of mineral P and K fertilizers were adjusted during the long-term experiment based on the soil chemical analyses. However, all the evaluated treatments always received the same doses of P and K fertilizers. Mineral N was applied as lime ammonium nitrate, P was applied as superphosphate, and K was applied as potassium chloride. All the mineral fertilizers were spread manually. The doses of mineral N were divided throughout the season, depending on the type of treatment. In autumn, before the wheat was sown, 40 kg ha^−1^ of N was applied as part of the N1PK, N2PK, and N3PK treatments. Another 40 kg ha^−1^ of N was applied at the beginning of spring for regeneration as part of the N2PK and N3PK treatments. Additionally, 40 kg ha^−1^ of N was applied in May to support grain production as part of the N3PK treatment. The seeding rate was typically 400 germinating grains m^−2^, with a row spacing of 0.125 m. Wheat sowing was usually conducted in September or at the beginning of October.

A total of 28 seasons are evaluated in this paper: 1979, 1980, 1981, 1982, 1991, 1992, 1993, 1994, 1995, 1996, 1997, 1998, 1999, 2000, 2001, 2002, 2003, 2004, 2005, 2006, 2011, 2012, 2013, 2014, 2015, 2016, 2017, and 2018. Over the course of the trial, three different preceding crops were used before planting wheat: (1) legumes (11 seasons), (2) cereals (13 seasons), and (3) oil plants (4 seasons). Together, seven wheat varieties were used during the analyzed period (1979–2022): (1) Slávia (1979–1982), (2) Hana (1991–1994), (3) Vega (1995–1998), (4) Brea (1999–2002), (5) Contra (2003–2006), (6) Mulan (2011–2014), and (7) Julie (2015–2022). All analyzed wheat varieties were of the short-straw variety.

In 2018, we decided to modify the long-term trial by increasing the mineral N rates. Between 2019 and 2022 (four seasons), the N rates in treatments N1PK, N2PK, and N3PK were increased to 60, 100, and 140 kg ha^−1^ N, respectively. The results from this time period were evaluated separately and used to determine the recommended mineral N rate for the specific soil and climate conditions. The preceding crop was legumes in this period.

### 4.2. Data Analysis

The results of the wheat grain yield as affected by the fertilizer treatment and preceding crop were checked using Shapiro-Wilk [101] and Anderson-Darling tests for normality distribution. The effect of the fertilizer treatments and preceding crops on wheat grain yield was analyzed using the Kruskal-Wallis one-way ANOVA, followed by the Conover-Iman post hoc test [102]. A parametric MANOVA (Hotelling-Lawley’s test) was used to evaluate the effect of year and fertilizer treatment on grain yield (the sample size is satisfactory for the parametric method). The grain yield and weather development (mean temperature and precipitation) were analyzed using the Mann-Kendall trend test [103,104] along with Sen’s slope estimation [105]. The homogeneity test was used for the selection of two time periods with different mean temperatures. For N dose optimization, a linear plateau response model was used. All analyses and graphical outputs were performed using Statistica 14.0 (Tibco Software, Palo Alto, CA, USA), SigmaPlot 14.5 (Systat Software Inc., San Jose, CA, USA) and XLStat software (Lumivero, Burlington, MA, USA).

## 5. Conclusions

The weather changed significantly at the long-term experimental site in Lukavac. It warmed up. The average annual increase in mean temperature was 0.035 °C, and the trend was statistically significant. The precipitation also slightly increased (1.163 mm per year), but the trend was not statistically significant. The weather conditions significantly affected the yield of winter wheat grain. In Lukavec, the main factors were the average temperature in February (r = −0.4) and the amount of precipitation in February (r = −0.4), March (r = −0.4), and May (r = 0.5). Nitrogen plays an indispensable role in plant nutrition, and its effect is very visible on naturally impoverished soils. In Lukavec, the yields increased linearly throughout time, particularly due to the use of modern wheat varieties and with increasing doses of mineral N. This relationship was particularly evident in the 1979–2018 period when the highest yields were achieved with the N3PK treatment, and all the fertilizer treatments were significantly different from each other. After the change in the methodology of the long-term experiment in 2018, when mineral N rates were increased, the statistical significance between the fertilization treatments started to blend. Both the N3PK and N2PK treatments provided statistically similar yields. This allowed for the utilization of a non-parametric response model, which determined an optimal N rate of 131 kg ha^−1^. This rate corresponds to an average yield of 8.2 t ha^−1^ when wheat is cultivated after legumes. Legumes were also evaluated as the most suitable preceding crops. In contrast, the lowest yields were recorded when wheat followed cereals.

## Figures and Tables

**Table 1:** Winter wheat mean grain yield (t ha^−1^) was analyzed in two periods: 1979–2018 (n = 28 seasons; middle column) and 2019–2022 (n = 4 seasons; right column; increased doses of mineral N fertilizers).

Fertilizer Treatment (kg ha^−1^)	Grain Yield (1979–2018)	Grain Yield (2019–2022)
Control	2.9 ± 1.2 A	4.2 ± 1.2 A
PK	3.5 ± 1.4 B	5.3 ± 1.4 AB
N1PK	4.6 ± 1.4 C	6.5 ± 1.7 BC
N2PK	5.7 ± 1.5 D	7.3 ± 1.4 CD
N3PK	6.6 ± 1.5 E	8.2 ± 1.2 D

Mean grain yields (±standard deviation of the mean) followed by the same letter are not statistically significantly different (α < 0.05).

**Table 2:** Winter wheat grain yield (t ha^−1^) as affected by wheat varieties used between 1979–2018 (n = 28 seasons).

Variety	Grain Yield (t ha^−1^)	Minimum	Maximum
Slávia	4.3 ± 1.2 B	2.0	6.4
Hana	4.4 ± 2.1 B	1.1	8.8
Vega	3.9 ± 1.6 AB	1.3	7.1
Brea	3.3 ± 1.3 A	1.6	6.1
Contra	5.8 ± 1.6 C	3.2	9.0
Mulan	6.2 ± 1.9 C	2.6	10.1
Julie (2015–2018)	4.7 ± 2.0 B	1.9	8.9
Julie (2019–2022)	6.3 ± 2.0 C	2.3	9.7

Mean grain yields (±standard deviation of the mean) followed by the same letter are not statistically significantly different (α < 0.05).

**Table 3:** The influence of the preceding crop on wheat grain yield (t ha^−1^).

Preceding Crop	Grain Yield (t ha^−1^)
Cereals	4.1 ± 1.8 A
Oil plants	4.7 ± 2.0 B
Legumes	5.4 ± 1.8 C

Mean grain yields (±standard deviation of the mean) followed by the same letter are not statistically significantly different (α < 0.05).

## Data Availability

The original contributions presented in the study are included in the article, further inquiries can be directed to the corresponding author/s.
